# Enhanced antimycobacterial efficacy of simulated inhaled clofazimine versus oral clofazimine in combination with azithromycin and ethambutol in a hollow-fiber system

**DOI:** 10.1093/jac/dkaf402

**Published:** 2025-11-10

**Authors:** Jelmer Raaijmakers, Rob Aarnoutse, Lindsey te Brake, Ralf Stemkens, Heiman Wertheim, Wouter Hoefsloot, Jakko van Ingen

**Affiliations:** Radboudumc Community for Infectious Diseases, Research Institute for Medical Innovation, Department of Medical Microbiology, Radboud University Medical Center, PO Box 1, Nijmegen 6500 HB, The Netherlands; Radboudumc Community for Infectious Diseases, Research Institute for Medical Innovation, Department of Pharmacy, Radboud University Medical Center, Nijmegen, The Netherlands; Radboudumc Community for Infectious Diseases, Research Institute for Medical Innovation, Department of Pharmacy, Radboud University Medical Center, Nijmegen, The Netherlands; Radboudumc Community for Infectious Diseases, Research Institute for Medical Innovation, Department of Pharmacy, Radboud University Medical Center, Nijmegen, The Netherlands; Radboudumc Community for Infectious Diseases, Research Institute for Medical Innovation, Department of Medical Microbiology, Radboud University Medical Center, PO Box 1, Nijmegen 6500 HB, The Netherlands; Radboudumc Community for Infectious Diseases, Research Institute for Medical Innovation, Department of Pulmonary Diseases, Radboud University Medical Center, Nijmegen 6500 HB, The Netherlands; Radboudumc Community for Infectious Diseases, Research Institute for Medical Innovation, Department of Medical Microbiology, Radboud University Medical Center, PO Box 1, Nijmegen 6500 HB, The Netherlands

## Abstract

**Background and objectives:**

Treatment outcomes in *Mycobacterium avium* complex pulmonary disease may be improved by adding clofazimine, instead of rifampicin, to the azithromycin-ethambutol backbone. Inhalation of clofazimine instead of oral administration has been suggested to increase the antibiotic concentration at the site of infection and to improve its efficacy, while minimizing systemic exposure and adverse effects. We evaluated the efficacy of inhaled clofazimine compared to oral clofazimine with an azithromycin-ethambutol backbone against *M. avium*.

**Methods:**

We simulated pharmacokinetic exposures to azithromycin, ethambutol and either inhalational or oral clofazimine administration in an *in vitro* hollow-fiber system during 3 weeks. Intracellular and extracellular *Mycobacterium avium* ATCC 700898 bacteria were exposed to these antibiotic regimens and bacterial densities were enumerated at day 0, 3, 7, 14 and 21. The development of macrolide resistance was assessed by inoculation of agar plates containing azithromycin. Pharmacokinetic exposures were confirmed on day 0 and 21.

**Results:**

Inhalational administration of clofazimine significantly increased the antimycobacterial effect of the regimen against both intracellular and extracellular bacteria. The inhaled treatment showed an intracellular kill rate of 0.62 (95%C.I. 0.61–0.64) per day, while the oral administration showed a kill rate of 0.55 (95%C.I. 0.54–0.56) per day. For the extracellular fraction, inhaled administration showed a kill rate of 0.56 (95%C.I. 0.55–0.58) per day and the oral administration a kill rate of 0.50 (95%C.I. 0.50–0.51) per day. Inhaled clofazimine exposures reduced and delayed the emergence of macrolide resistance.

**Conclusions:**

Inhalation of clofazimine with an azithromycin-ethambutol backbone increases treatment efficacy and decreases the development of macrolide resistance compared to oral administration in a hollow-fiber system. This calls for a clinical trial of inhaled clofazimine.

## Introduction

Treatment of *Mycobacterium avium* complex pulmonary disease (MAC-PD) has high failure and recurrence rates.^1^ The recommended antibiotic treatment is a rifampicin, ethambutol and azithromycin combination regimen,^1^ but the role of rifampicin has been disputed as it lacks *in vitro* activity.^2^ Clofazimine has been suggested as a replacement of rifampicin since the clofazimine-based regimen proved more effective in the hollow-fiber pharmacodynamic model.^3^ A randomized clinical trial showed similar effectiveness of clofazimine- and rifampicin-containing regimens. In this trial clofazimine plasma concentrations were higher among patients with culture conversion, compared to those without culture conversion.^4^ This suggests that higher exposures may increase the efficacy of clofazimine.

There may be a role for clofazimine to improve MAC-PD treatment outcomes, but its dose and associated exposure first need to be optimized.^5^ The current oral dose is 100 mg/day,^1,4,6^ but doses as high as 300 mg/day have been used long-term in leprosy treatment and were re-evaluated as loading doses for NTM disease.^5,7^ Another way of optimizing local clofazimine exposure and effect in MAC-PD would be to use an inhaled formulation. Inhaled clofazimine proved efficacious in mouse models of MAC-PD,^8^ safe in dogs^9^ and a human trial is planned (ClinicalTrials.gov ID: NCT06418711).

Here we evaluate the administration of inhaled clofazimine versus oral clofazimine with a backbone of azithromycin and ethambutol in an Hollow-fiber system *Mycobacterium avium* (HFS-MAC) model of pulmonary disease.

## Materials and methods

### Bacteria, cells and antibiotics

The *Mycobacterium avium* ATCC 700898 reference strain was obtained from the American Type Culture Collection (ATCC, Manassas, VA) and stored at −20°C. Prior to experimentation, the strain was cultured in Middlebrook 7H9 medium (Becton Dickinson, Vianen, The Netherlands) supplemented with 5% OADC (Becton Dickinson, Vianen, The Netherlands) under conditions of 36°C and 5% CO_2_ for five days. THP-1 cells were sourced from the German Collection of Microorganisms and Cell Cultures (DSMZ; Braunschweig, Germany; ACC 16 Lot 32) and stored at −180°C. Upon thawing, the THP-1 cells were washed and cultured in RPMI 1640 (VWR International, Amsterdam, The Netherlands) supplemented with 20% foetal bovine serum (FBS; Life Technologies Limited, Paisley, UK). After two passages, the concentration of FBS was reduced to 10%.

Stock solutions of azithromycin, ethambutol, and clofazimine (Sigma Aldrich, Zwijndrecht, The Netherlands) were prepared in ethanol, Milli-Q water, and DMSO, respectively. Azithromycin and ethambutol syringe solutions were formulated by diluting the stock solutions to a 40/60% (v/v) ratio of ethanol to Milli-Q water and in Milli-Q water, respectively (see Table S4 (available as Supplementary data at *JAC* Online)). Additionally, a bolus solution of clofazimine at 0.1 mg/mL was prepared in RPMI 1640 supplemented with 2% FBS, 10% DMSO, and 0.5% Tween 80.

### MIC determinations

The minimum inhibitory concentrations (MIC) of azithromycin, ethambutol and clofazimine against *M. avium* ATCC 700898 were determined according to CLSI guidelines before and after the experiment to detect development of drug tolerance.^10^

### Hollow-fiber setup

The hollow-fiber systems were assembled as previously described.^3^ Briefly, cellulosic hollow-fiber cartridges (C8008, FiberCell Systems, New Market, MD, USA) were first rinsed with Milli-Q water for 24 hours, followed by washing with RPMI 1640 supplemented with 2% FBS. Clofazimine binding sites were then saturated as outlined in the Supplementary Materials (see Chapter S6 (available as Supplementary data at *JAC* Online)).

Each hollow-fiber system (HFS) had a total volume of distribution of 370 mL, which included 300 mL in the central reservoir, 20 mL in the tubing, and 50 mL in the hollow-fiber cartridge. The diluent was stored in large 4-liter bottles, and the flow rate into the central reservoir was set to 0.4 mL/min to align with the half-life of ethambutol. Differences in half-life were corrected for by a zero-order top-up of azithromycin. Meanwhile, the flow rate from the central reservoir to the elimination reservoir was adjusted to 2.5 mL/min to prevent overflow of the central reservoir.

Prior to initiating the experiment, THP-1 cells (representing human macrophages) were prepared at a density of 2 × 10^6^ cells/mL in RPMI 1640 supplemented with 10% heat-inactivated FBS. These cells were infected with a 0.5 McFarland suspension of *M. avium* and incubated for 12 hours at 36°C in a 5% CO_2_ environment, in a bacteria-to-cell ratio of 1:3. The infected THP-1 cells were seeded into the hollow-fiber cartridge four hours before the experimental start.

### Hollow-fiber study design and simulated pharmacokinetic profiles

The HFS-MAC was run for a total duration of 21 days in triplicate, with drug-free growth controls, oral clofazimine treatment and inhaled clofazimine treatment, both combined with azithromycin and ethambutol.

We simulated unbound epithelial lining fluid pharmacokinetics (ELF) of daily doses of azithromycin (250 mg) and ethambutol (15 mg/kg) as described earlier, see Table 1 for parameter values.^3^ One deviation was in the simulated T_max_ for azithromycin, which was 4 hours.^11^ Pharmacokinetic targets of azithromycin and ethambutol were the same in both therapies. Oral intrapulmonary pharmacokinetics of clofazimine (100 mg/day) were simulated as described before, targeting a C_avg_ of 2.2 mg/L after oral clofazimine administration.^3^ A daily flat dose of 80 mg for inhaled clofazimine was simulated, in line with a clinical trial registered on clinicaltrials.gov (NCT06418711). The effectively delivered dose (30% of the total dose)^13^ was divided by the alveolar volume of distribution to determine the target C_max_ after a single dose. To estimate the alveolar distribution volume, we calculated the total human alveolar surface area and multiplied it by the thickness of the epithelial lining fluid (see Table S1). This yielded an alveolar region volume of 21 mL,^14,15^ which is in line with previously reported volumes.^16^ Assuming 99% binding to cellular components for clofazimine in the lung after oral administration,^3^ it was reasonable to apply the same assumption to the inhaled administration. By multiplying the dose with both the effectively administered fraction and the protein binding percentage, we derived the amount of free clofazimine available within the volume of distribution, leading to a target C_max_ of 11.4 mg/L (see formula F1, the solved formula F2, and Chapter S1).


(F1) $$C_{max}=\frac{Dose\cdot fraction\;deposited\cdot protein\;binding}{lung\;volume}$$

(F2) $$C_{max}=\frac{80\cdot 0.3\cdot 0.01}{0.021}=11.43\;mg/L$$

clofazimine cannot penetrate the cellulosic membranes of the hollow-fibre cartridge to reach the extra-capillary space, and was manually dosed in the extra-capillary space as a bolus every day. Due to the long clofazimine half-life (±80 days) *in vivo*, we aimed for a static lung clofazimine concentration in the HFS.^7^ Clofazimine is unstable in RPMI1640 + 2% FBS, displaying a degradation half-life of 14 hours with first-order kinetics. Therefore, a C_max_ of 4 mg/L was targeted for oral clofazimine administration to reach an average concentration of 2.2 mg/L. For inhaled clofazimine (C_max_ target 11.4 mg/L), the expected average concentration was 6.7 mg/L. Drug-cartridge compatibility testing results are shown in Chapters S3 and S4.

**Table 1.** Target pharmacokinetic parameters and actual attained parameters in the hollow-fiber system at steady-state

			Oral CFZ	Inhaled CFZ
Parameter	Drug	Target	Actual (± S.E.)	Actual (± S.E.)
T_1/2_ (h)	Azithromycin	20^11^	19.8 ± 3.5	18.9 ± 1.9
	Ethambutol	10^12^	12.0 ± 1.0	11.5 ± 0.7
T_max_ (h)	Azithromycin	4^11^	4.0 ± 0.0	4.0 ± 0.0
	Ethambutol	3^12^	3.3 ± 0.5	3.0 ± 0.0
C_max,ss_ (mg/L)	Azithromycin	3.75^11^	4.7 ± 0.45	4.4 ± 0.2
	Ethambutol	3^12^	3.9 ± 0.04	4.0 ± 0.1
	Oral CFZ	4	3.9 ± 0.4	—
	Inhaled CFZ	11.4	—	10.2 ± 1.5
AUC_0–24_ (mg·h/L)	Azithromycin	62.0	69.5 ± 4.2	65.8 ± 3.8
	Ethambutol	48.5	52.9 ± 1.1	55.7 ± 1.5
C_avg_ (mg/L)	Oral CFZ	2.2	2.4 ± 0.1	—
	Inhaled CFZ	6.7	—	5.9 ± 0.5

Values are geometric means of the three values (± standard error).

CFZ, clofazimine.

### Pharmacokinetic measurements

On days 0 and 21, complete pharmacokinetic curves of azithromycin and ethambutol were generated, and concentrations were analysed using ultra-high-performance liquid chromatography-mass spectrometry as previously described (see chapter S7).^3^ For azithromycin and ethambutol, samples were collected from the central reservoir at time points T = 0, 1.5, 3, 4, 5, 7, 9, 11, 13, 15, and 24 hours, and processed immediately to stabilize the antibiotics. All samples were measured simultaneously after the sampling day. To assess clofazimine exposure, at day 0 and 21, samples were taken pre-dose from the hollow-fiber cartridge at T = 0 (T_max_), as well as at 12 and 24 hours. The area under the concentration-time curve (AUC_0–24_) was calculated and divided by 24 hours to obtain the average concentration (C_avg_).

### Bacterial and THP-1 cell enumerations

Pharmacodynamic samples were collected from the extra-capillary space on days 0, 3, 7, 14, and 21 (see Chapter S7). For each sample, 1 mL was centrifuged at 1500 RPM for 10 minutes. The supernatant was used to enumerate the extracellular bacterial fraction, while the pellet was lysed in 1 mL of Milli-Q water with 0.05% Tween 80 (Sigma Aldrich, Zwijndrecht, Netherlands) to quantify the intracellular fraction. Both fractions were serially diluted 10-fold, and three 10 µL drops of each dilution were plated on Middlebrook 7H10 agar (Becton Dickinson, Vianen, Netherlands) and on Middlebrook 7H10 agar containing 8× the initial MIC of azithromycin to identify subpopulations with reduced macrolide susceptibility due to increased tolerance. Additionally, unprocessed pharmacodynamic samples were diluted 1:1 with trypan blue reagent (Sigma Aldrich, Zwijndrecht, Netherlands) and analysed using a KOVA counting grid to determine THP-1 cell densities.

### Calculations & statistics

To assess pharmacodynamic effects, colony-forming units (cfu) per mL were log_10_-transformed and plotted against time using GraphPad Prism 7.0.0 (GraphPad Software Inc., La Jolla, CA, USA). The net bacterial growth rate (K_net_) and the maximum bacterial carrying capacity (B_max_) for both intracellular and extracellular fractions were estimated by fitting a growth model to the raw data, as described in formula (F3), utilizing the R package NLMIXR. Subsequently, the parameters derived from the growth model were incorporated and fixed (K_net,fix_ and B_max,fix_) into an antibiotic effect model (F4), in which an antibiotic effect parameter was included as a decrease in K_net_. The antibiotic effect parameter is the kill rate (cfu/day) achieved in the HFS cartridge.


(F3) $$\frac{d}{dt}B=K_{net}\cdot \left(1-\frac{B}{B_{\max}}\right)\cdot B$$

(F4) $$\frac{d}{dt}\;B=(K_{net,fix}-Antibiotic\;effect)\cdot \left(1-\;\frac{B}{B_{max,fix}}\right)\cdot B$$

To evaluate the pharmacokinetics, antibiotic concentrations of azithromycin and ethambutol were plotted against time to generate complete pharmacokinetic curves using GraphPad prism 7.0.0 (GraphPad Software Inc., LA Jolla, CA, USA). Non-compartmental analysis of pharmacokinetic curves was performed on azithromycin and ethambutol curves using Phoenix 64 WinNonlin (Build 8.3.1.5.014). The linear-up log-down trapezoidal rule was used to calculate the elimination rate constant *λz* and the subsequent half-life of the drugs (T_1/2_ = 0.693/*λz*). For the calculation of *λz*, at least five measured concentrations were analysed after the C_max_. The clofazimine AUC_0–24_ were divided by 24 hours to calculate the mean concentrations.

## Results

### Pharmacokinetic evaluation

Azithromycin and ethambutol had similar exposures in both arms and fulfilled the preset target 24-hour exposure (Table 1). Complete pharmacokinetic curves can be found in the Supplementary Materials (Chapter S2). The oral clofazimine exposure in the HFS-MAC was close to the target level. For the inhaled route of clofazimine administration, a 10% lower C_max_ than targeted was realized (Table 1).

### Minimum inhibitory concentrations

Pretreatment MICs of *M. avium* ATCC 700898 for azithromycin, ethambutol and clofazimine were 8, 8 mg/L and 0.12 mg/L, respectively, and had not increased at the end of the experiment.

### Pharmacodynamic effect and emergence of macrolide resistance

**Figure 1.**
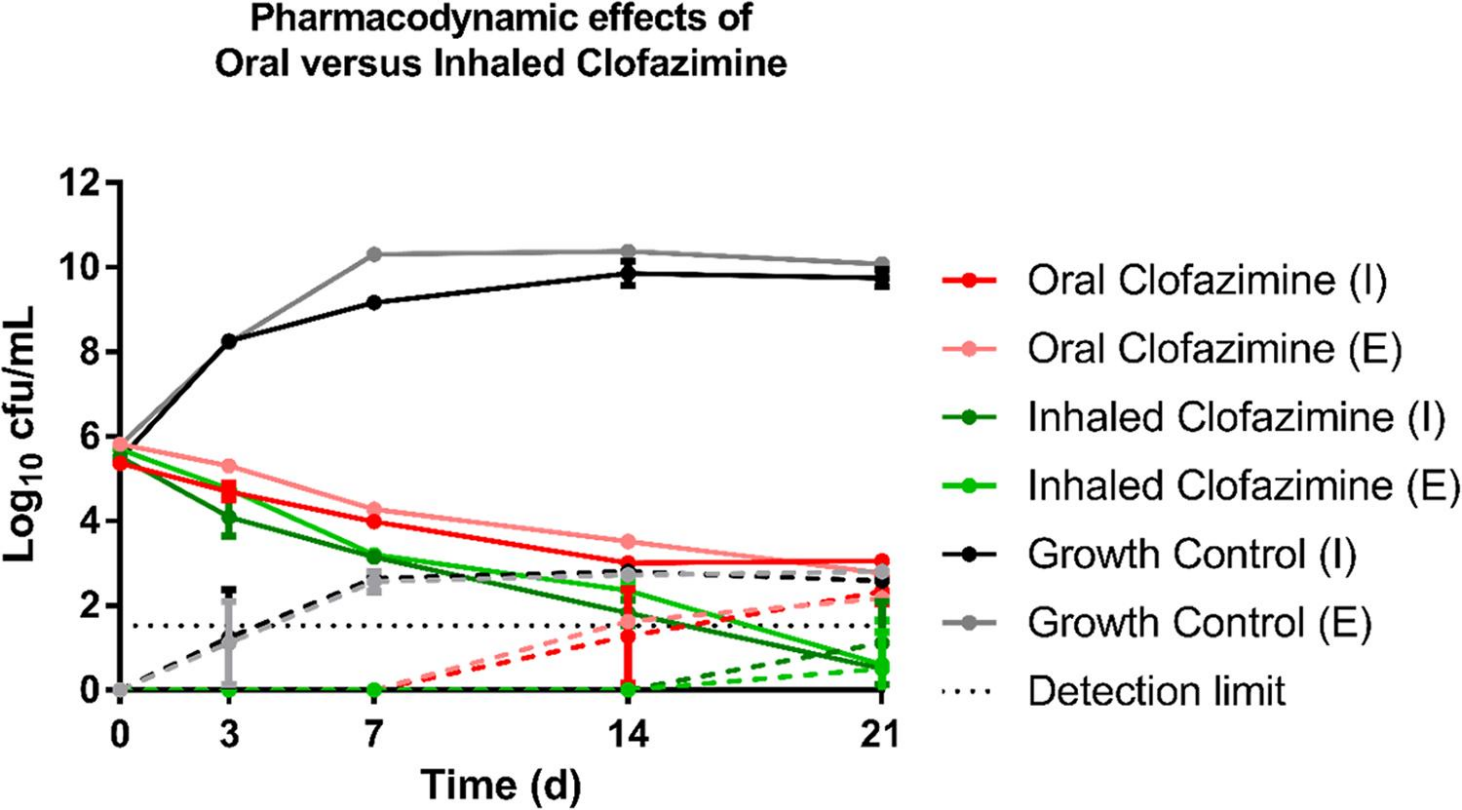
Hollow fiber pharmacodynamic effects (mean and error bars) of orally administered clofazimine and inhaled clofazimine (with a backbone of azithromycin and ethambutol) and unexposed growth controls. (I) represents the intracellular fraction. (E) represents the extracellular fraction. Lines connect log_10_ cfu/mL measurements. The dashed lines represent the density of M. avium subpopulations within the hollow-fibre system that exhibit tolerance to azithromycin at concentrations 8× the initial MIC.

Exposures associated with inhalational administration of clofazimine significantly increased the antimycobacterial effect of the treatment against both intracellular and extracellular *M. avium* (Figure 1). The inhaled clofazimine treatment showed an intracellular kill rate of 0.62 (95% C.I. 0.61–0.64), while the oral formulation showed a kill rate of 0.55 (95% C.I. 0.54–0.56). For the extracellular fraction, inhaled administration showed a kill rate of 0.56 (95% C.I. 0.55–0.58) and the oral formulation a kill rate of 0.50 (95% C.I. 0.50–0.51). The inhaled clofazimine formulation delayed and reduced the extent of the emergence of macrolide tolerance (Figure 1; dashed lines).

### THP-1 cell densities

THP-1 cells were replenished every other day to counteract the bacterial cell death induced by the administration of DMSO and the thorough mixing of the cartridge contents.^3^ The THP-1 cell densities remained stable throughout the experiment and are presented in the Supplementary Materials (Chapter S5).

## Discussion

In the hollow fiber system, clofazimine exposures attainable with inhalation significantly improved the pharmacodynamic effects of the regimen over that of oral administration of a standard dose of clofazimine in conjunction with azithromycin and ethambutol against *Mycobacterium avium*. Additionally, the findings indicate a delayed onset of macrolide-tolerance and a reduction in macrolide-tolerant subpopulation sizes. Importantly, there were no observed changes in the minimum inhibitory concentrations (MICs) for any of the three drugs utilized, indicating that there was no significant development of irreversible drug tolerance.

A clear exposure-dependent effect is observed with increasing clofazimine concentrations, considering that the expected and simulated clofazimine ELF concentrations after inhaled administration were higher than those after oral administration (Table 1). The exposure-dependent effect of clofazimine has been assumed before, but was not clearly proven, based on animal models.^5,8^ An upcoming clinical trial (clinicaltrials.gov NCT06418711) will use a daily dose of 80 mg of inhaled clofazimine in NTM patients, with a month on/month off schedule. Animal studies in mice^8^ and beagles^9^ have shown that inhaled doses up to 10 mg/kg and 2.72 mg/kg, respectively, were well tolerated, suggesting that even higher exposures than the 80 mg daily dose in humans might be tolerable and may further increase the antimycobacterial effect of clofazimine.

The pharmacodynamic effect observed in the inhalational clofazimine administration arm may be underestimated, as we did not simulate clofazimine accumulation in the HFS-MAC due to the unknown specifics of its pharmacokinetics related to accumulation following inhalation. The activity of oral clofazimine exposures was similar to a previous HFS-MAC study.^3^ Higher exposures for better effect might not require inhalation; the safety and pharmacokinetics of higher oral doses are also being investigated and have been studied in the past.^5^ The dose used in this study is sufficient to elicit a robust antibacterial effect, and that the inhalation route may offer flexibility in dosing without compromising therapeutic outcomes.

A limitation of the current study is that the clofazimine inhalation concentration was derived from a theoretical framework rather than clinical studies measuring intrapulmonary concentrations following inhalation. Available pharmacokinetic data from animal studies—specifically in mice^8^ and Beagle dogs^9^ —may not accurately reflect human pharmacokinetics. Additionally, *in vitro* models like the HFS, have inherent limitations. In this study, we exposed the reference strain of *M. avium* to the treatment regimens; however, due to intra- and inter-individual variability in drug exposure and the broad diversity of mycobacterial species infecting patients, variations in responses are expected.

In summary, this HFS-MAC study provides promising evidence that an increased exposure of clofazimine, as will be achieved via inhalation, further enhances the antimycobacterial efficacy of ethambutol and azithromycin against *M. avium* in both intracellular and extracellular environments, more so than oral clofazimine in its current dosing. The enhanced kill rates observed in the HFS-MAC led to reduced bacterial concentrations, which in a clinical context would translate to a lower overall bacterial burden with likely decreased inflammation and potentially improved treatment outcome. It also shows potential to inhibit the development of macrolide tolerance. Inhalational administration of clofazimine may therefore improve treatment outcomes for MAC-PD patients. Further clinical investigation is warranted to confirm these findings and optimize dosing strategies for human use.

## Supplementary Material

dkaf402_Supplementary_Data

## Supplementary data

Tables S1 to S22 and Chapters S1 to S8 is available as Supplementary data at *JAC* Online.
